# Prognostic Value of Strain by Speckle Tracking Echocardiography in Patients with Arrhythmogenic Right Ventricular Cardiomyopathy

**DOI:** 10.3390/jcdd11120388

**Published:** 2024-12-03

**Authors:** Areej Aljehani, Kyaw Zaw Win, Shanat Baig, Manish Kalla, Bode Ensam, Larissa Fabritz, Richard P. Steeds

**Affiliations:** 1 Institute of Cardiovascular Sciences, University of Birmingham, Birmingham B15 2TT, UK; axa1388@student.bham.ac.uk (A.A.); k.z.win@bham.ac.uk (K.Z.W.); shanat.baig@uhb.nhs.uk (S.B.); manish.kalla@uhb.nhs.uk (M.K.); bode.ensam@uhb.nhs.uk (B.E.); l.fabritz@bham.ac.uk (L.F.); 2 Department of Cardiology, University Hospitals Birmingham NHS Foundation Trust, Birmingham B15 2GW, UK; 3 Echocardiography Cardiovascular Technology (ECVT) Program, King Saud bin Abdulaziz University for Health Sciences, Riyadh 11481, Saudi Arabia; 4 University Center for Cardiovascular Science & Department of Cardiology, University Heart and Vascular Center, University Hospital Hamburg-Eppendorf, Martinistrasse 52, 20246 Hamburg, Germany; 5 German Center for Cardiovascular Research (DZHK), Partner Site Hamburg/Kiel/Lübeck, Martinistrasse 52, 20246 Hamburg, Germany

**Keywords:** arrhythmogenic right ventricular cardiomyopathy, transthoracic echocardiography, speckle tracking, global longitudinal strain, prognosis

## Abstract

Background Arrhythmogenic right ventricular cardiomyopathy (ARVC) is a rare genetic disorder associated with an elevated risk of life-threatening arrhythmias and progressive ventricular impairment. Risk stratification is essential to prevent major adverse cardiac events (MACE). Our study aimed to investigate the incremental value of strain measured by two-dimensional speckle-tracking echocardiography in predicting MACE in ARVC patients compared to conventional echocardiographic parameters. Methods and Results This was a retrospective, single-centre cohort study of 83 patients with ARVC (51% males, median age 37 years (IQR: 23, 53)) under the care of the Inherited Cardiac Conditions clinic at University Hospital Birmingham. MACE was defined as one of the following: sustained ventricular tachycardia (Sus VT), ventricular fibrillation (VF), appropriate implantable cardio-defibrillator (ICD) therapy [shock/anti-tachycardia pacing (ATP)], heart failure (defined as decompensated heart failure, cardiac index by heart catheter, HF medication, and symptoms), cardiac transplantation, or cardiac death. Echocardiography images were analysed by a single observer for right ventricle (RV) and left ventricular (LV) global longitudinal strain (GLS). Multivariable Cox regression was performed in combination with RV fractional area change and tricuspid annular plane systolic excursion. During three years of follow-up, 12% of patients suffered a MACE. ARVC patients with MACE had significantly reduced RV GLS (−13 ± 6% vs. −23 ± 6%, *p* < 0.001) and RV free wall longitudinal strain (−15 ± 5% vs. −25 ± 7%, *p* < 0.001) compared to those without MACE. Conclusions Right ventricular free wall longitudinal strain (RVFWLS) may be a more sensitive predictor of MACE than conventional echocardiographic parameters of RV function. Moreover, RV-free wall longitudinal strain may have superior predictive value compared to RV GLS.

## 1. Introduction

Arrhythmogenic right ventricular cardiomyopathy (ARVC) is an inherited cardiac disease that is the primary cause of sudden cardiac death in individuals under the age of 35 [1]. Histopathologically, the disease is characterised by the replacement of cardiomyocytes with fibrofatty tissue, mainly in the right ventricle (RV), leading to structural and functional abnormalities [2]. Accurate evaluation of the risk of arrhythmia is critical since arrhythmias can manifest in the initial stages of the disease (before many of the diagnostic structural changes are evident) [3], and prompt implantation of a cardioverter-defibrillator (ICD) can save lives [4]. Echocardiography plays a key role in the diagnosis and clinical evaluation of patients with ARVC, serving as a valuable method for detecting right ventricle (RV) abnormalities and assessing prognosis in this group of patients [5,6]. Speckle tracking echocardiography (STE) has emerged as a more accurate measurement of RV dysfunction, even when conventional parameters remain within the normal range [7,8].

RV strain can be analysed either as RV global longitudinal strain (RVGLS), which includes the interventricular septum, or RV free wall longitudinal strain (RVFWLS), excluding the interventricular septum. Both techniques provide different perspectives on RV myocardial deformation. Although both ventricles share the same myocardial fibres within the interventricular septum, it is considered part of the left ventricle (LV) and mainly reflects the movement of the LV [9]. A comparison between both techniques in terms of their predictive value for adverse events holds significant clinical importance. To date, there has been no previous comparison of RVGLS and RVFWLS to determine which RV strain parameter may be superior for predicting cardiac events among patients with ARVC.

Therefore, this study aimed to (1) assess whether STE-RV strain can predict major adverse cardiac events (MACE); (2) evaluate the incremental value of STE-RV strain over conventional echocardiographic parameters in ARVC patients without prior MACE; (3) compare the predictive value of RVFWLS and RVGLS in patients with ARVC.

## 2. Methods

### 2.1. Study Population

This was a retrospective study that recruited patients from a single centre, the Inherited Cardiac Conditions clinic (ICC) at the University Hospitals Birmingham, United Kingdom. The study included adult patients aged 18 and older with definite and non-definite (early) ARVC from 2010 to 2022. Patients were excluded if they had hypertension, ischemia, or valve disease as the most likely cause of heart disease. Patients were also excluded if they had suffered a MACE prior to entry. Demographics, medical history, and clinical data were identified through the interrogation of digital health records.

The categorisation of patients into definite and non-definite (early) ARVC was based on the 2010 Task Force Criteria (TFC). A definite ARVC diagnosis was made when two major criteria or one major and two minor criteria or four minor criteria from different categories were met. A non-definite ARVC diagnosis was made when one major and one minor criterion or three minor criteria from different categories were met or when one major or two minor criteria from different categories were met [3]. This determination was made through a discussion by a multidisciplinary team, including ICC clinicians with expertise in electrophysiology, genetics, and cardiovascular imaging. Non-definite ARVC patients (borderline and possible) who had not undergone genetic testing were excluded because they might not represent true early ARVC cases. Definite ARVC patients who did not undergo genetic testing but exhibited clear ARVC phenotypes were included, however, as their clinical presentation met the diagnostic criteria.

### 2.2. Electrocardiographic Evaluation

In accordance with our previous published studies of patients within the ICC clinic [10,11], all patients underwent a 12-lead ECG, signal-averaged ECG (SAECG) and 24 h ambulatory ECG. Phenotypic ECG criteria were assessed in accordance with the 2010 TFC [3].

### 2.3. Echocardiography

Transthoracic echocardiography (TTE) was conducted by qualified echocardiographers accredited by the British Society of Echocardiography (BSE). Images were reanalysed offline using IntelliSpace Cardiovascular technology (ISCV; Philips, Amsterdam, The Netherlands) by a single observer (AA). Conventional parameters were measured in accordance with the latest requirements outlined in the established BSE guidelines [12]. RV fractional area change (RV-FAC) was calculated from the RV end-diastolic area (RVEDA) and RV end-systolic area (RVESA), measured in the apical four-chamber view. Tricuspid annular plane systolic excursion (TAPSE) was measured by M-mode, also in the apical four-chamber view. LV ejection fraction (LV-EF) was obtained using the biplane Simpson method. 3D volumetry was not available for clinical studies during the time of recruitment.

RV and LV strains were assessed using two-dimensional speckle tracking echocardiography (2DSTE) by one independent operator (AA) using the Tomtec Imaging System. LVGLS was obtained by averaging values from the four, three, and two-chamber views by automatically tracking the endocardial borders during end-diastole and end-systole. Global longitudinal strain was not calculated in those cases where regional tracking was poor in 2 or more myocardial segments. RV strain was obtained in the apical four-chamber view. Endocardial borders were automatically tracked during end-diastole and end-systole, with manual corrections if necessary. The RV was then automatically divided into six segments, which included basal, middle, and apical segments of both the interventricular septum and RV free wall. RVGLS was obtained by averaging values of the six segments, while RVFWLS was obtained by averaging values of the three segments of the RV free wall.

The first available echocardiographic images were taken at the time of the examination. In cases where multiple examinations were available, we consistently chose the first trackable echocardiogram to ensure uniformity in our data analysis while maintaining the best possible image quality for accurate assessment.

The primary outcome of this study was the occurrence of MACE following echocardiography. MACE was defined as one of the following: sustained ventricular tachycardia (VT), ventricular fibrillation (VF), appropriate implantable cardio-defibrillator (ICD) therapy [shock/anti-tachycardia pacing (ATP)], heart failure (defined as decompensated heart failure, impaired cardiac index by heart catheter, HF medication, and symptoms), cardiac transplantation, or death. Outcome data were collected via an exhaustive review of individual electronic patient records. As per standard clinical practice, clinical episodes occurring elsewhere were reported to the clinical team at University Hospitals Birmingham NHS Foundation Trust, and a comprehensive review of clinical episodes was performed as part of the standard follow-up.

## 3. Statistical Analysis

Continuous variables are presented as mean (±standard deviation) for normally distributed variables or median (interquartile range) for non-normally distributed variables and compared using independent-sample *t*-tests or Mann–Whitney U tests, respectively. Categorical variables are presented as proportions (%) and compared using Fisher’s exact tests or Chi-squared tests. Differentiation between normally and non-normally distributed data was assessed by the Shapiro–Wilk test. Statistical analyses were performed using IBM SPSS 27. To determine the association between the strain and the primary outcome, univariate and multivariable Cox regression analyses were performed. Patients were censored at the date of their last follow-up or the date of the first MACE event, whichever occurred first. To ensure that our data were not influenced by overfitting, we added each strain variable to separate models in combination with (i) RV-FAC, (ii) TAPSE and (iii) LVGLS.

## 4. Results

### 4.1. Patient Cohort

**Figure 1:**
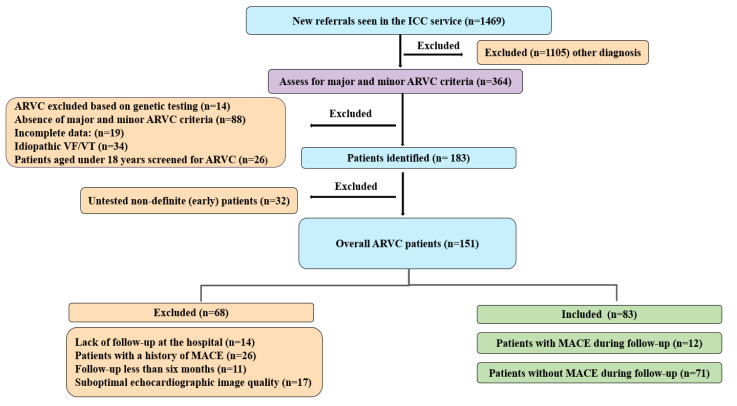
Patients referred to UHB with definite and non-definite ARVC.

As demonstrated in Figure 1, during the period from 2010 to 2022, the ICC clinic received 1469 new patients for confirmed or suspected arrhythmia syndromes, including ARVC. Out of these, 1105 patients were ruled out due to a different diagnosis. The most common categories included long QT syndrome, hypertrophic cardiomyopathy (HCM), dilated cardiomyopathy (DCM), Brugada syndrome, and catecholaminergic polymorphic ventricular tachycardia (CPVT). A total of 364 patients were specifically assessed for the presence of major and minor ARVC criteria as per the 2010 TFC. Of these, 181 patients were excluded for the following reasons: negative genetic testing for family members of an ARVC proband with a confirmed pathogenic variant (n = 14), patients who did not meet the criteria for either a definite or non-definite diagnosis of ARVC (n = 88), incomplete phenotypic data (n = 19), patients aged under 18 years screened for ARVC (n = 26), and those with idiopathic VF/VT (n = 34). Of the remaining 183 patients, 32 non-definite (early) patients were excluded for not undergoing genetic testing. The total number of ARVC patients was 151. Of those, 68 patients were excluded for various reasons, including lack of follow-up at the hospital (n = 14), patients with a prior history of MACE (n = 26), those with follow-up less than six months (n = 11), and patients with suboptimal echocardiography image quality (n = 17). The final study population included 83 patients, consisting of 12 patients with MACE during follow-up and 71 patients without MACE.

### 4.2. Baseline Characteristics

Of the 83 ARVC patients diagnosed according to the 2010 TFC, 42 (51%) subjects were male, with a median age of 37 (IQR: 23, 53) years, and most carried a pathogenic gene variant associated with ARVC 58 (71%). Patients with MACE tended to fulfil a definite ARVC diagnosis at baseline (n = 9 (75%) vs. n = 17 (24%), *p* = 0.001), experienced more syncope (n = 7 (58%) vs. n = 3 (4%), *p* < 0.001), and palpitations (n = 9 (75%) vs. n = 17 (24%), *p* = 0.001) at inclusion. There was no difference in median age (43 (IQR: 32, 52) vs. 37 (IQR: 23, 53) years, *p* = 0.641) or sex (male n = 7 (58%) vs. male n = 35 (49%), *p* = 0.756) and the presence of a pathogenic variant was not associated with MACE (n = 7 (58%) vs. n = 51 (73%), *p* = 0.320). During a median follow-up of 3 (IQR 1, 5) years, twelve (14%) patients developed MACE, including six patients with sustained VT, three patients who received ICD shocks, and three patients who developed HF. The baseline characteristics of the study subjects are shown in Table 1.

### 4.3. Electrocardiographic Evaluation

The 12-lead ECG, SAECG and 24 h ambulatory ECG data from patients with and without MACE are summarised in the Supplementary Table S1. Epsilon waves were noted only in the three patients (25%) with MACE during follow-up, while patients without MACE demonstrated no major ECG depolarisation criteria (*p* < 0.001). Minor ECG repolarisation criteria were more commonly observed in patients with MACE (n = 7 (58%) vs. n = 5 (7%), *p* < 0.001). Additionally, patients with MACE showed a higher prevalence of PVCs during a 24 h ambulatory ECG recording (n = 6 (86%) vs. n = 9 (30%), *p* = 0.011). 

### 4.4. Imaging Evaluation

**Figure 2:**
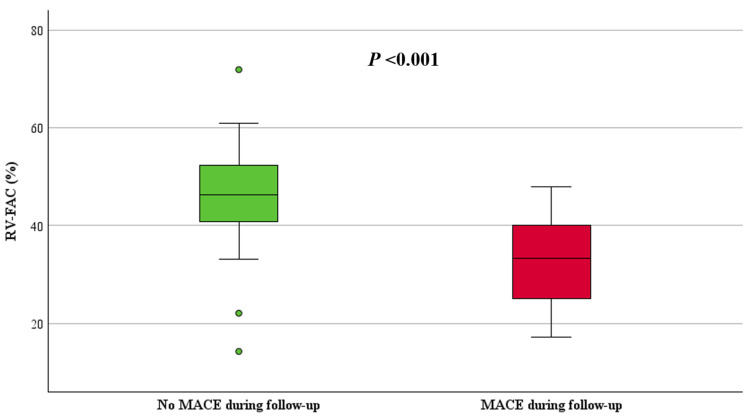
Boxplot of RV-FAC in patients with and without MACE.

**Figure 3:**
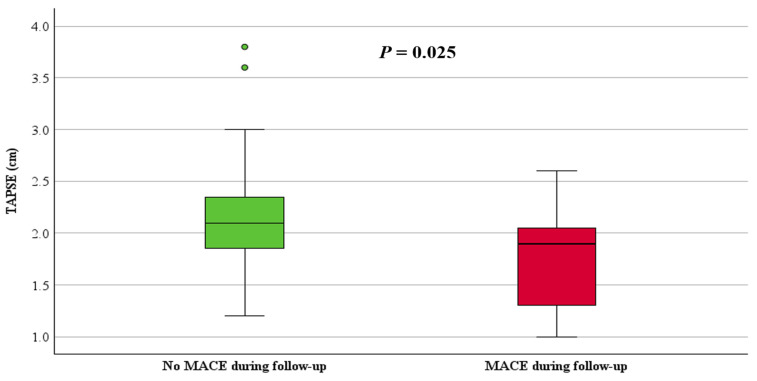
Boxplot of TAPSE in patients with and without MACE.

ARVC patients with MACE had significantly reduced right ventricular function, with a median RV-FAC of 33% (IQR: 25, 40) compared to 46% (IQR: 41, 53, *p* < 0.001) in those without MACE (Figure 2). Additionally, TAPSE was 1.9 cm (IQR: 1.3, 2.1) in patients with MACE and 2.1 cm (IQR: 1.8, 2.4, *p* = 0.025) in those without MACE (Figure 3). The RVOT diameter by PLAX view was dilated in patients with MACE (3.8 ± 0.6 cm vs. 2.8 cm ± 0.6, *p* < 0.001) compared to those without MACE. Moreover, patients with MACE during follow-up showed significantly lower LV-EF 57% (IQR: 48, 62) vs. 62% (IQR: 58, 67, *p* = 0.027) and presented with a greater extent of LGE (n = 6 (86%) vs. n = 10 (25%), *p* = 0.004) compared to those without MACE (Table 2). Figure S1 in the supplementary illustrates the RV and LV-LGE distribution in patients with and without MACE.

### 4.5. Strain Analysis

**Figure 4:**
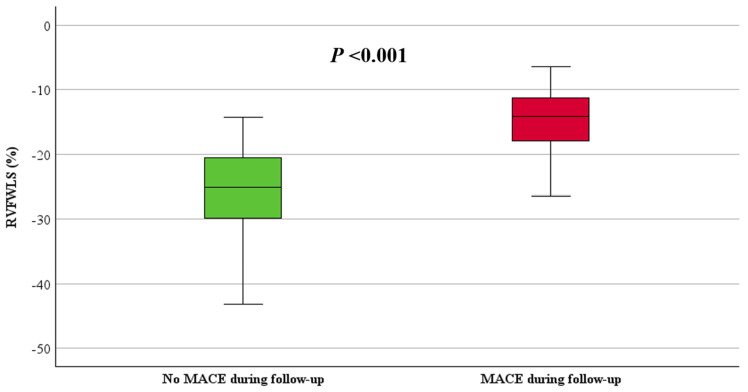
Boxplot of RVFWLS in patients with and without MACE.

**Figure 5:**
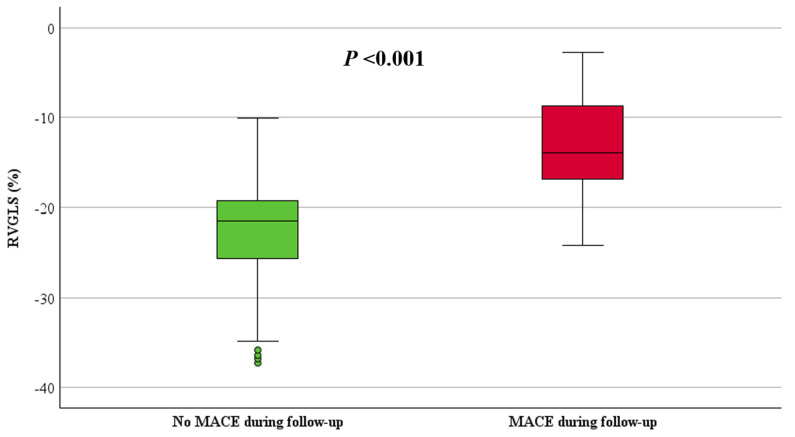
Boxplot of RVGLS in patients with and without MACE.

**Figure 6:**
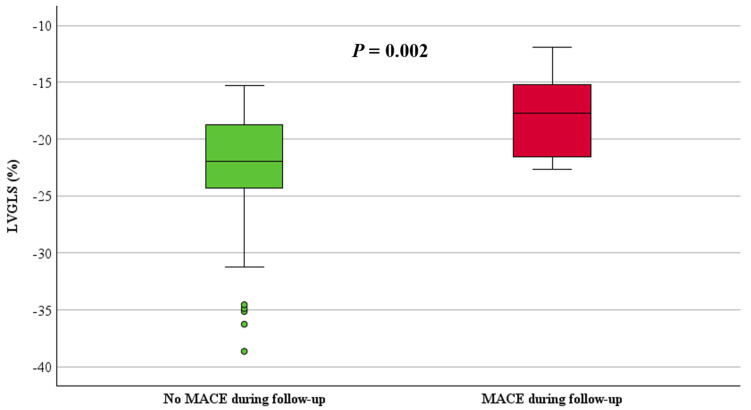
Boxplot of LVGLS in patients with and without MACE.

RVFWLS and RVGLS, stratified by the occurrence of MACE, are shown in Table 2. Both RVFWLS and RVGLS were significantly reduced in patients with MACE compared to those without MACE (−15 ± 5% vs. −25 ± 7%, *p* < 0.001 and −13 ± 6% vs. −23 ± 6%, *p* < 0.001, respectively), (Figure 4 and Figure 5). Additionally, LV GLS values were significantly decreased in patients with MACE compared to those without MACE (−18 ± 4% vs. −23 ± 5%, *p* = 0.002), (Figure 6).

To assess the predictive value of RV-STE over conventional parameters, we performed univariate and multivariable Cox regression analyses for RVFWLS and RVGLS in combination with (i) RV-FAC, (ii) TAPSE, and (iii) LVGLS, as demonstrated in Table 3 and Table 4. All RV functional parameters were strongly associated with MACE in univariate Cox regression analyses. However, RVFWLS remained the only significant predictor in multivariate analyses when compared to RV-FAC (HR 1.4, 95% CI 1.0–2.0, *p* = 0.031) and TAPSE (HR 1.4, 95% CI 1.1–2.0, *p* = 0.020). Furthermore, LVGLS was a significant predictor of MACE during follow-up (HR 1.3, 95% CI 1.0–1.7, *p* = 0.034), as shown in Table 4. After identifying outliers in our data, we conducted a separate analysis excluding these outliers. Remarkably, the results remained consistent, indicating that the presence of outliers did not significantly impact our findings; the results are shown in Supplementary Tables S2 and S3.

Additionally, a separate analysis was performed that only included definite patients. The results from both univariate and multivariate Cox regression analyses showed that only LV GLS remained a significant predictor of MACE. These results are shown in Supplementary Tables S4 and S5.

To assess the predictive value between RVFWLS and RVGLS, a separate Cox regression model was performed (Table 4, model 4). RVFWLS demonstrated a stronger predictive value for MACE (HR 1.4, 95% CI 1.0–2.0, *p* = 0.026) compared to RVGLS (HR 0.9, 95% CI 0.7–1.2, *p* = 0.377), as shown in Table 4. In addition, the predictive value of segmental strain was assessed, but only the apical RVFWLS segment was predictive of MACE, as shown in Supplementary Table S6.

## 5. Discussion

The major findings of our study were that ARVC patients with lower RV strain, including both RVGLS and RVFWLS, have a greater risk of MACE over an average follow-up of 3 years. After adjustment for TAPSE and RV-FAC, only RVFWLS showed incremental value over conventional echocardiographic parameters in predicting adverse events.

Our data found that strain, as measured by RVGLS, RVFWLS, and LVGLS, is associated with MACE during follow-up in ARVC patients. Several reports have shown the value of RV strain in ARVC patients [13,14,15]. Sarvari et al. [16] reported on the utility of RV strain in detecting asymptomatic mutations in individuals prone to ventricular arrhythmias. Furthermore, Kirkkles et al. [13] validated these results in a larger cohort consisting of 150 definite ARVC patients and emphasised the utility of RV strain in identifying patients at risk of arrhythmia. We found that RVFWLS was the only significant predictor of MACE after correcting for RV-FAC and TAPSE. Previous studies have evaluated the performance of multiple RV strain parameters and their relationship with ventricular arrhythmia [17,18,19] as well as the utility of conventional echocardiographic parameters, particularly RV-FAC [20], in relation to ventricular arrhythmia. Lie et al. [18] demonstrated that RV longitudinal strain (expressed as mechanical dispersion) was associated with ventricular arrhythmias in ARVC patients during follow-up. Moreover, Kirkels et al. [19] found that 29% (47/170) of ARVC patients with a history of life-threatening ventricular arrhythmias demonstrated longer RV mechanical dispersion compared to those without (53 ms vs. 21 ms, *p* < 0.001). More importantly, they found that adding RV mechanical dispersion to RV deformation patterns categorised by the sub-tricuspid region significantly improved the association with ventricular arrhythmias in ARVC patients. While these results may seem promising, it is important to note that these studies did not compare the predictive value of RV strain and conventional parameters.

In our study, Cox regression analysis demonstrated that RVFWLS has superior value over traditional functional assessments (RV-FAC and TAPSE) in predicting those patients at risk of MACE. This could be attributed to the fact that RV longitudinal strain provides continuous evaluation of myocardial function by tracking acoustic markers (called speckles) throughout the cardiac cycle [21], allowing identification of both peak contraction and maximum relaxation. In contrast, RV-FAC assesses frames only at end-systole and end-diastole, offering a limited snapshot of ventricular function at these particular phases. TAPSE has several limitations, including angle and load dependency, along with the possible confounding impact of left ventricular function [22]. Further, assessing the structure and function of the RV by echocardiography poses challenges due to the intricate geometry of the RV [16]. Therefore, incorporating RV-free wall strain into routine echocardiographic assessment in ARVC patients might prove beneficial for several reasons. Firstly, strain measurements offer higher sensitivity as they can detect early myocardial changes [23], often before conventional methods. Secondly, RV-free wall strain provides additional value in predicting outcomes such as the risk of arrhythmia and heart failure [24]. This information enables more tailored and proactive patient management, specifically influencing the timing of ICD implantation. In addition, RV-free wall strain is easier to evaluate and widely available compared to other strain variables, such as mechanical dispersion and sub-tricuspid patterns.

LVGLS is associated with a worse prognosis in various cardiovascular conditions [25,26,27]. In our study, we found that LVGLS predicted patients at risk of MACE. This is to be expected, as LV function remains a key factor in identifying high-risk patients for potential ICD implantation in primary prevention [28], and LVGLS has superior value to LV-EF in predicting cardiovascular outcomes [29]. Including LVGLS in the echocardiography assessment of ARVC patients is important for a more comprehensive evaluation and early detection of LV involvement, which should improve risk stratification and enhance patient care.

While progress is being made in standardising RV strain measurements, there is an ongoing debate regarding the clinical utility of measuring RVGLS, which includes both the interventricular septum and the RV free wall, versus RVFWLS, which excludes the interventricular septum. Previous studies comparing RVGLS and RVFWLS are conflicting, with a prospective observational study [30] showing that RVGLS appears to outperform RV free wall GLS in predicting mortality in patients undergoing cardiac resynchronisation therapy. Nevertheless, it should be emphasised that that study did not include left ventricular function as a predictor in Cox regression analysis. In contrast, Gao Y et al. [31] compared the prognostic value of both techniques in patients with repaired tetralogy of Fallot during 2.8 years of follow-up and observed that RVFWLS was identified as a more accurate predictor of adverse outcomes compared to both RV and LV GLS. Although the findings of that study align with our observations, the results needed confirmation due to the different populations recruited. Therefore, our data showed that RVFWLS exhibits superior performance compared to RV GLS. This could be explained by the involvement of the interventricular septum in RV GLS, which is more susceptible to influence from LV movement [32]. Furthermore, RVFWLS specifically evaluates the function of the RV-free wall, which is often the most affected area in ARVC pathology; focusing on this region may provide a precise tool for risk stratification.

## 6. Limitations

We only included patients with no history of MACE, and caution should be exercised when applying our findings to those with a previous MACE (secondary prevention). In this paper, ATP was included as a component of MACE, as we felt it was important to capture episodes of VT that may reflect electrical instability due to the arrhythmogenic substrate and also as a potential marker of so-called ‘hot phases’. We acknowledge, however, that ATP alone may not be a definitive predictor of SCD. Strain analysis was not available for all patients due to suboptimal image quality. SAECG and 24 Holter were not performed for all patients, and some were not conducted at baseline; however, they were performed as close as possible to the time of admission. The small number of MACE restricts the validity of the analysis, but this is an inevitable limitation to any study of rare diseases. Acknowledging this limitation, our results are consistent with previously published cohorts, and we believe they add to the literature on the utility of strain in ARVC.

## 7. Conclusions

Our study found that RVFWLS is a strong predictor of adverse events, showing incremental value over conventional parameters and RVGLS and contributing to more accurate risk stratification in ARVC patients. Future studies might focus on exploring the predictive value of 3D STE in assessing cardiovascular outcomes in this patient population.

## Figures and Tables

**Table 1:** Clinical and demographic characteristics of the study cohort.

Demographic	Overall = 83	MACE During Follow-Up = 12	No MACE During Follow-Up = 71	*p* Value
Age (years), median (IQR)	37 (23, 53)	43 (32, 52)	37 (23, 53)	0.641
Male sex, (%)	42 (51%)	7 (58%)	35 (49%)	0.756
Body surface area (m^2^), median (IQR)	1.9 (1.7, 2.0)	1.9 (1.7, 2.0)	1.9 (1.7, 2.0)	0.466
**Stage of ARVC**				
Definite, (%)	26 (31%)	9 (75%)	17 (24%)	0.001
Early, (%)	57 (69%)	3 (25%)	54 (76%)	
^a^ Pathogenic variant (%)	58 (71%)	7 (58%)	51 (73%)	0.320
PKP2, (%)	32 (39%)	4 (33%)	28 (40%)	0.757
DSP, (%)	25 (30%)	2 (17%)	23 (33%)	0.328
DSG2, (%)	1 (1%)	1 (8%)	0 (0%)	0.146
Variant of unknown significance (VUS), (%)	1 (1%)	1 (8%)	0 (0%)	0.146
No pathogenic mutation identified, (%)	23 (28%)	4 (33%)	19 (27%)	0.731
**Symptoms**				
Palpitation, (%)	26 (31%)	9 (75%)	17 (24%)	0.001
Syncope, (%)	10 (12%)	7 (58%)	3 (4%)	<0.001
^b^ **History of sport**				
History of Competitive sport, (%)	15 (20%)	4 (36%)	11 (17%)	0.302
History of Non-competitive sport, (%)	14 (19%)	1 (9%)	13 (21%)	
No history of sport, (%)	45 (61%)	6 (55%)	39 (62%)	
**Medication**				
Statins, (%)	4 (5%)	3 (25%)	1 (1%)	0.009
Anticoagulant, (%)	5 (6%)	3 (25%)	2 (3%)	0.020
Antiarrhythmic drugs, (%)	5 (6%)	3 (25%)	2 (3%)	0.020
Beta blocker, (%)	11 (13%)	6 (50%)	5 (7%)	<0.001
Follow-up period (years), median (IQR)	3 (1, 5)	1 (1, 5)	3 (2, 5)	0.123

Data are reported as median (IQR), with *p*-values from Mann–Whitney U tests; or as number (%), with *p*-values from Fisher’s exact tests. ^a^ genetic testing was performed of 82 patients, ^b^ sport information was available for 74 patients.

**Table 2:** Imaging characteristics of patients with and without MACE during follow-up.

	Overall = 83	MACE During Follow-Up = 12	No MACE During Follow-Up = 71	*p* Value
**Echo Data**				
RVOT PLAX (cm), mean ± SD	3.0 ± 0.7	3.8 ± 0.6	2.8 ± 0.6	<0.001
RVEDA (cm^2^), median, (IQR)	19 (16, 24)	27 (20, 35)	18 (15, 21)	<0.001
RV-FAC (%), median, (IQR)	45 (39, 51)	33 (25, 40)	46 (41, 53)	<0.001
TAPSE (cm), median, (IQR)	2.0 (1.8, 2.3)	1.9 (1.3, 2.1)	2.1 (1.8, 2.4)	0.025
LVEDV (ml), mean ± SD	98 ± 30	99 ± 42	98 ± 28	0.926
LV-EF (%), median, (IQR)	61 (57, 66)	57 (48, 62)	62 (58, 67)	0.027
STE-RVFWLS (%), mean ± SD	−24 ± 8	−15 ± 5	−25 ± 7	<0.001
STE-RVGLS (%), mean ± SD	−21 ± 7	−13 ± 6	−23 ± 6	<0.001
STE-LVGLS (%), mean ± SD	−22 ± 5	−18 ± 4	−23 ± 5	0.002
^a^ LGE present, (%)	16 (34%)	6 (86%)	10 (25%)	0.004
RV-LGE, (%)	11 (23%)	4 (57%)	7 (18%)	0.042
LV-LGE, (%)	10 (21%)	4 (57%)	6 (15%)	0.029

Abbreviations: RVOT PLAX: right ventricular outflow tract parasternal long axis; RVEDA: right ventricular end-diastolic area; RV-FAC: right ventricular fractional area change; TAPSE: tricuspid annular plane systolic excursion; RVFWLS: right ventricular free wall longitudinal strain; RVGLS: right ventricular global longitudinal strain; LVGLS: left ventricular global longitudinal strain; LGE: late gadolinium enhancement. ^a^ LGE assessment was performed in 48 patients (with MACE n = 7, no MACE n = 40).

**Table 3:** Univariable Cox proportional hazards model for MACE prediction of right ventricular global longitudinal strain (RVGLS) and right ventricular free wall longitudinal strain (RVFWLS).

	Univariable Model	
	HR (95% CI)	*p*-Value
RV-FAC (%)	0.9 (0.9, 1.0)	0.004
TAPSE (cm)	0.1 (0.0, 0.7)	0.015
STE-RVFWLS (%)	1.3 (1.1, 1.4)	<0.001
STE-RVGLS (%)	1.2 (1.1, 1.3)	<0.001
STE-LVGLS (%)	1.5 (1.2, 1.9)	0.002

Abbreviations: RV-FAC: right ventricular fractional area change; TAPSE: tricuspid annular plane systolic excursion; RVFWLS: right ventricular free wall longitudinal strain; RVGLS: right ventricular global longitudinal strain; LVGLS: left ventricular global longitudinal strain.

**Table 4:** Multivariable Cox proportional hazards model for MACE prediction.

	Multivariable Model 1		Multivariable Model 2		Multivariable Model 3		Multivariable Model 4	
	RV-FAC		TAPSE		STE-LVGLS			
	HR (95% CI)	*p*-Value	HR	*p*-Value	HR	*p*-Value	HR	*p*-Value
			(95% CI)		(95% CI)		(95% CI)	
RV-FAC (%), per unit measure	1.0 (0.9, 1.1)	0.926	-	-	-	-	-	-
TAPSE (cm), per unit measure	-	-	0.5 (0.1, 2.5)	0.367	-	-	-	-
STE-RVFWLS (%), per unit measure	1.4 (1.0, 2.0)	0.031	1.4 (1.1, 2.0)	0.020	1.6 (1.1, 2.4)	0.010	1.4 (1.0, 2.0)	0.026
STE-RVGLS (%) per unit measure	0.9 (0.7,1.2)	0.379	0.9 (0.6, 1.1)	0.243	0.7 (0.5, 1.0)	0.060	0.9 (0.7, 1.2)	0.377
STE-LVGLS (%) per unit measure	-	-	-	-	1.3 (1.0, 1.7)	0.034		

Abbreviations: STE: speckle tracking echocardiography; RV-FAC: right ventricular fractional area change; TAPSE: tricuspid annular plane systolic excursion; RVFWLS: right ventricular free wall longitudinal strain; RVGLS: right ventricular global longitudinal strain; LVGLS: left ventricular global longitudinal strain.

## Data Availability

The anonymised datasets used and/or analysed during the current study are available from the corresponding author upon reasonable request.

## Supplementary Materials

The following supporting information can be downloaded at: https://www.mdpi.com/article/10.3390/jcdd11120388/s1, Figure S1: Right ventricular (RV) and left ventricular (LV) LGE distribution on CMR in patients with and without MACE during follow-up; Figure S2: ROC curve, including all ARVC patients; Table S1: Electrical characteristics of patients with and without MACE during follow-up; Table S2: Univariable Cox proportional hazards model for MACE prediction after excluding outliers; Table S3: Multivariable Cox proportional hazards model for MACE prediction after excluding outliers; Table S4: Univariable Cox proportional hazards model for MACE prediction in definite patients; Table S5: Multivariable Cox proportional hazards model for MACE prediction in definite patients; Table S6: Univariable and multivariable Cox proportional hazards model for MACE prediction for segmental strain; Table S7: Cut-off values for strain determined using ROC curve analysis.

## Abbreviations

ARVC: arrhythmogenic right ventricular cardiomyopathy; VA: ventricular arrhythmias; SCD: sudden cardiac death; VF: ventricular fibrillation; MACE: major adverse cardiac event; HF: heart failure; RVGLS: right ventricular global longitudinal strain; RVFWLS: right ventricular free wall longitudinal strain; ECG: electrocardiogram; SAECG: signal-averaged ECG; TWI: T wave inversion; RBB: right bundle branch block; PVC: premature ventricular contractions; ICD: implantable cardioverter-defibrillator; LGE: late gadolinium enhancement; LV: left ventricle; RV: right ventricle; SCD: sudden cardiac death; TFC: task force criteria; RVOT: right ventricle outflow tract; PLAX; parasternal long axis; FAC: fractional area change; RVEDA: right ventricular end-diastolic area; RV-FAC: right ventricular fractional area change; LVEDD: left ventricular end-diastolic volume; LV-EF: left ventricular ejection fraction; STE: speckle tracking echocardiography.

## References

[1] Jin Q., Lee K.Y., Selimi Z., Shimura D., Wang E., Zimmerman J.F., Shaw R.M., Kucera J.P., Parker K.K., Saffitz J.E. (2024). Determinants of electrical propagation and propagation block in Arrhythmogenic Cardiomyopathy. J. Mol. Cell. Cardiol..

[2] Corrado D., Link M.S., Calkins H. (2017). Arrhythmogenic right ventricular cardiomyopathy. N. Engl. J. Med..

[3] Marcus F.I., McKenna W.J., Sherrill D., Basso C., Bauce B., Bluemke D.A., Calkins H., Corrado D., Cox M.G., Daubert J.P. (2010). Diagnosis of arrhythmogenic right ventricular cardiomyopathy/dysplasia: Proposed modification of the task force criteria. Circulation.

[4] Corrado D., Wichter T., Link M.S., Hauer R.N., Marchlinski F.E., Anastasakis A., Bauce B., Basso C., Brunckhorst C., Tsatsopoulou A. (2015). Treatment of arrhythmogenic right ventricular cardiomyopathy/dysplasia: An international task force consensus statement. Circulation.

[5] Aneq M.Å., Lindström L., Fluur C., Nylander E. (2008). Long-term follow-up in arrhythmogenic right ventricular cardiomyopathy using tissue Doppler imaging. Scand. Cardiovasc. J..

[6] Sadeghpour A., Hosseini L., Rezaeian N., Alizadehasl A., Maleki M., Emkanjoo Z., Bakhshandeh H., Zadehbagheri F. (2020). Presence and prognostic value of ventricular diastolic function in arrhythmogenic right ventricular cardiomyopathy. Echocardiography.

[7] Kamal N.M., Salih A.F., Ali B.M. (2024). Speckle tracking echocardiography for diagnosis of right ventricular failure in children with totally corrected tetralogy of Fallot in Sulaimani, Iraq. J. Taibah Univ. Med. Sci..

[8] Badano L.P., Muraru D. (2016). Subclinical right ventricular dysfunction by strain analysis: Refining the targets of echocardiographic imaging in systemic sclerosis. Am. Heart Assoc..

[9] Ho S.Y. (2009). Anatomy and myoarchitecture of the left ventricular wall in normal and in disease. Eur. J. Echocardiogr..

[10] Aljehani A., Kew T., Baig S., Cox H., Sommerfeld L., Ensam B., Kalla M., Steeds R., Fabritz L. (2023). Characterisation of patients referred to a tertiary-level inherited cardiac condition clinic with suspected arrhythmogenic right ventricular cardiomyopathy (ARVC). BMC Cardiovasc. Disord..

[11] Aljehani A., Baig S., Kew T., Kalla M., Sommerfeld L.C., Murukutla V.A., Fabritz L., Steeds R.P. (2024). Structural Progression in Patients with Definite and Non-Definite Arrhythmogenic Right Ventricular Cardiomyopathy and Risk of Major Adverse Cardiac Events. Biomedicines.

[12] Robinson S., Rana B., Oxborough D., Steeds R., Monaghan M., Stout M., Pearce K., Harkness A., Ring L., Paton M. (2020). A practical guideline for performing a comprehensive transthoracic echocardiogram in adults: The British Society of Echocardiography minimum dataset. Echo Res. Pract..

[13] Kirkels F.P., Rootwelt-Norberg C., Bosman L.P., Aabel E.W., Muller S.A., Castrini A.I., Taha K., van Osta N., Lie Ø.H., Asselbergs F.W. (2023). The added value of abnormal regional myocardial function for risk prediction in arrhythmogenic right ventricular cardiomyopathy. Eur. Heart J.-Cardiovasc. Imaging.

[14] Claeys M., Claessen G., Claus P., De Bosscher R., Dausin C., Voigt J.-U., Willems R., Heidbuchel H., La Gerche A. (2020). Right ventricular strain rate during exercise accurately identifies male athletes with right ventricular arrhythmias. Eur. Heart J.-Cardiovasc. Imaging.

[15] Sarvari S.I., Haugaa K.H., Anfinsen O.-G., Leren T.P., Smiseth O.A., Kongsgaard E., Amlie J.P., Edvardsen T. (2011). Right ventricular mechanical dispersion is related to malignant arrhythmias: A study of patients with arrhythmogenic right ventricular cardiomyopathy and subclinical right ventricular dysfunction. Eur. Heart J..

[16] Friedberg M.K. (2021). Peeking Beyond Strain’s Peak: Regional Strain Patterns and Dispersion in Arrhythmogenic Right Ventricular Cardiomyopathy.

[17] Leren I.S., Saberniak J., Haland T.F., Edvardsen T., Haugaa K.H. (2017). Combination of ECG and echocardiography for identification of arrhythmic events in early ARVC. JACC Cardiovasc. Imaging.

[18] Lie Ø.H., Rootwelt-Norberg C., Dejgaard L.A., Leren I.S., Stokke M.K., Edvardsen T., Haugaa K.H. (2018). Prediction of life-threatening ventricular arrhythmia in patients with arrhythmogenic cardiomyopathy: A primary prevention cohort study. JACC Cardiovasc. Imaging.

[19] Kirkels F.P., Lie Ø.H., Cramer M.J., Chivulescu M., Rootwelt-Norberg C., Asselbergs F.W., Teske A.J., Haugaa K.H. (2021). Right ventricular functional abnormalities in arrhythmogenic cardiomyopathy: Association with life-threatening ventricular arrhythmias. Cardiovasc. Imaging.

[20] Saguner A.M., Vecchiati A., Baldinger S.H., Rüeger S., Medeiros-Domingo A., Mueller-Burri A.S., Haegeli L.M., Biaggi P., Manka R., Lüscher T.F. (2014). Different prognostic value of functional right ventricular parameters in arrhythmogenic right ventricular cardiomyopathy/dysplasia. Circ. Cardiovasc. Imaging.

[21] Roy C., Duclos G., Nafati C., Gardette M., Lopez A., Pastene B., Gaudray E., Boussuges A., Antonini F., Leone M. (2021). Left ventricular longitudinal strain variations assessed by speckle-tracking echocardiography after a passive leg raising maneuver in patients with acute circulatory failure to predict fluid responsiveness: A prospective, observational study. PLoS ONE.

[22] DiLorenzo M.P., Bhatt S.M., Mercer-Rosa L. (2015). How best to assess right ventricular function by echocardiography. Cardiol. Young.

[23] Thavendiranathan P., Poulin F., Lim K.-D., Plana J.C., Woo A., Marwick T.H. (2014). Use of myocardial strain imaging by echocardiography for the early detection of cardiotoxicity in patients during and after cancer chemotherapy: A systematic review. J. Am. Coll. Cardiol..

[24] Hamada-Harimura Y., Seo Y., Ishizu T., Nishi I., Machino-Ohtsuka T., Yamamoto M., Sugano A., Sato K., Sai S., Obara K. (2018). Incremental prognostic value of right ventricular strain in patients with acute decompensated heart failure. Circ. Cardiovasc. Imaging.

[25] Chimed S., Stassen J., Galloo X., Meucci M.C., Knuuti J., Delgado V., van der Bijl P., Marsan N.A., Bax J.J. (2023). Prognostic Relevance of Left Ventricular Global Longitudinal Strain in Patients with Heart Failure and Reduced Ejection Fraction. Am. J. Cardiol..

[26] Zhu D., Ito S., Miranda W.R., Nkomo V.T., Pislaru S.V., Villarraga H.R., Pellikka P.A., Crusan D.J., Oh J.K. (2020). Left ventricular global longitudinal strain is associated with long-term outcomes in moderate aortic stenosis. Circ. Cardiovasc. Imaging.

[27] Tower-Rader A., Betancor J., Popovic Z.B., Sato K., Thamilarasan M., Smedira N.G., Lever H.M., Desai M.Y. (2017). Incremental prognostic utility of left ventricular global longitudinal strain in hypertrophic obstructive cardiomyopathy patients and preserved left ventricular ejection fraction. J. Am. Heart Assoc..

[28] Segura-Rodríguez D., Bermúdez-Jiménez F.J., González-Camacho L., Moreno Escobar E., García-Orta R., Alcalá-López J.E., Bautista Pavés A., Oyonarte-Ramírez J.M., López-Fernández S., Álvarez M. (2021). Layer-specific global longitudinal strain predicts arrhythmic risk in arrhythmogenic cardiomyopathy. Front. Cardiovasc. Med..

[29] Smiseth O.A., Torp H., Opdahl A., Haugaa K.H., Urheim S. (2016). Myocardial strain imaging: How useful is it in clinical decision making?. Eur. Heart J..

[30] Nagy V.K., Széplaki G., Apor A., Kutyifa V., Kovács A., Kosztin A., Becker D., Boros A.M., Gellér L., Merkely B. (2015). Role of right ventricular global longitudinal strain in predicting early and long-term mortality in cardiac resynchronization therapy patients. PLoS ONE.

[31] Gao Y., Li H., He L., Zhang Y., Sun W., Li M., Gao L., Lin Y., Ji M., Lv Q. (2022). Superior prognostic value of right ventricular free wall compared to global longitudinal strain in patients with repaired tetralogy of Fallot. Front. Cardiovasc. Med..

[32] Lang R.M., Badano L.P., Mor-Avi V., Afilalo J., Armstrong A., Ernande L., Flachskampf F.A., Foster E., Goldstein S.A., Kuznetsova T. (2015). Recommendations for cardiac chamber quantification by echocardiography in adults: An update from the American Society of Echocardiography and the European Association of Cardiovascular Imaging. Eur. Heart J. Cardiovasc. Imaging.

