# Supplementary material for: Prognostic Value of Strain by Speckle Tracking Echocardiography in Patients with Arrhythmogenic Right Ventricular Cardiomyopathy
Source: J Cardiovasc Dev Dis. 2024 Dec 3;11(12):388. doi: 10.3390/jcdd11120388 (PMC11678278; doi:10.3390/jcdd11120388)
Supplement: Supplementary file 1 [file jcdd-11-00388-s001.zip › jcdd-3073863-supplementary.pdf]

Supplementary Online Content

Table S1. Electrical characteristics of patients with and without MACE during follow-up

Table S2. Univariable Cox proportional hazards model for MACE prediction after excluding outliers

Table S3. Multivariable Cox proportional hazards model for MACE prediction after excluding outliers

Table S4. Univariable Cox proportional hazards model for MACE prediction in definite patients

Table S5. Multivariable Cox proportional hazards model for MACE prediction in definite patients

Table S6. Univariable and multivariable Cox proportional hazards model for MACE prediction for segmental strain

Table S7. Cut-off values for strain determined using ROC curve analysis

Figure S1 Right ventricular (RV) and left ventricular (LV) LGE distribution on CMR in patients with and without MACE during follow-up

Figure S2. ROC curve, including all ARVC patients.

Table S1. Electrical characteristics of patients with and without MACE during follow-up

**Baseline ECG**	**Overall =83**	**MACE during follow-up= 12**	**No MACE during follow-up=71**	**P value**
Heart rate (bpm), mean ± SD	66 ± 12	65 ± 14	67 ± 12	0.698
PR interval (ms), mean ± SD	153 ± 23	165 ± 25	151 ± 22	0.102
QRS duration (ms), median, (IQR)	92 (86, 102)	101 (83, 111)	92 (86, 102)	0.299
**Depolarisation criteria**				
Major criteria, n (%), Epsilon wave in the right precordial leads (V1–V3) , n (%)	3 (4%)	3 (25%)	0 (0%)	**0.002**
[a] Minor criteria, n(%) Signal-averaged ECG with late potential (if QRS on standard surface <110 ms), n (%)	35 (48%)	6 (60%)	29 (46%)	0.505
**Repolarisation criteria**				
Major criteria, n (%), TWI in right precordial leads (V1, V2 and V3) , n (%)	13 (16%)	3 (25%)	10 (14%)	0.390
Any minor criteria, n (%), TWI in leads V1 and V2 or in V4, V5, and V6, TWI in leads V1, V2, V3, and V4 with RBBB, n (%)	12 (14%)	7 (58%)	5 (7%)	**<0.001**
[b] >500 PVC / 24 hours (Holter), n (%)	15 (41%)	6 (86%)	9 (30%)	**0.011**

Abbreviation: TWI: T wave inversion; RBB: right bundle branch block; BBB: bundle branch block; PVC: premature ventricular contractions.
[a] SAECG was performed in 73 patients (with MACE n=10, no MACE n=63), [b] 24 hours (Holter) was performed in 37 patients (with MACE n=7, no MACE n=30).

Table S2. Univariable Cox proportional hazards model for MACE prediction after excluding outliers

	Univariable model	
	**HR (95%CI)**	**P-value**
RV-FAC (%)	0.9 (0.9, 1.0)	**0.005**
TAPSE (cm)	0.1 (0.0, 0.6)	**0.012**
RVFWLS(%)	1.3 (1.1, 1.4)	**<0.001**
RVGLS (%)	1.2 (1.1, 1.3)	**<0.001**
LVGLS(%)	1.5 (1.2, 1.9)	**0.002**

Abbreviation: RVFAC: right ventricular fractional area change; TAPSE: tricuspid annular plane systolic excursion; RVFWLS: right ventricular free wall longitudinal strain; RVGLS: right ventricular global longitudinal strain; LVGLS: left ventricular global longitudinal strain.

Table S3. Multivariable Cox proportional hazards model for MACE prediction after excluding outliers

	**Multivariable model 1 RV-FAC**		**Multivariable model 2 TAPSE**		**Multivariable model 3 LVGLS**		**Multivariable model 4**	
	**HR (95%CI)**	**P-value**	**HR (95%CI)**	**P-value**	**HR (95%CI)**	**P-value**	**HR (95%CI)**	**P-value**
RV-FAC (%)	1.0 (0.9, 1.1)	0.931	-	-	-	-	-	-
TAPSE (cm)	-		0.5 (0.1, 2.5)	0.365	-	-	-	-
RVFWLS(%)	1.4 (1.0, 2.0)	**0.032**	1.4 (1.1, 2.0)	**0.020**	1.6 (1.1, 2.4)	**0.010**	1.4 (1.0, 2.0)	**0.026**
RVGLS (%)	0.9 (0.7, 1.2)	0.379	0.9 (0.6,1.1)	0.242	0.7 (0.5, 1.0)	0.060	0.9 (0.7, 1.2)	0.377
LVGLS(%)	-	-	-	-	1.3 (1.0, 1.7)	**0.034**	-	-

Abbreviation: RVFAC: right ventricular fractional area change; TAPSE: tricuspid annular plane systolic excursion; RVFWLS: right ventricular free wall longitudinal strain; RVGLS: right ventricular global longitudinal strain; LVGLS: left ventricular global longitudinal strain.

Table S4. Univariable Cox proportional hazards model for MACE prediction in definite patients

**Definite only**	**Univariable model**	
	**HR (95%CI)**	**P-value**
RV-FAC (%)	1.0 (0.9, 1.0)	0.148
TAPSE (cm)	0.2 (0.0, 1.4)	0.109
RVFWLS(%)	1.3 (1.1, 1.6)	**0.014**
RVGLS (%)	1.2 (1.1, 1.4)	**0.009**
LVGLS(%)	1.6 (1.1, 2.1)	**0.004**

Abbreviation: RVFAC: right ventricular fractional area change; TAPSE: tricuspid annular plane systolic excursion; RVFWLS: right ventricular free wall longitudinal strain; RVGLS: right ventricular global longitudinal strain; LVGLS: left ventricular global longitudinal strain.

Table S5. Multivariable Cox proportional hazards model for MACE prediction in definite patients

	**Multivariable model 1** RV-FAC		**Multivariable model 2** TAPSE		**Multivariable model 3** LVGLS		**Multivariable model 4**	
	**HR (95%CI)**	**P-value**	**HR (95%CI)**	**P-value**	**HR (95%CI)**	**P-value**	**HR (95%CI)**	**P-value**
RV-FAC (%)	-	-	-	-	-	-	-	-
TAPSE (cm)	-	-	-	-	-	-	-	-
RVFWLS (%)	-	-	-	-	1.8 (0.8, 3.9)	0.131	1.3 (0.8, 2.1)	0.333
RVGLS (%)	-	-	-		0.7 (0.4, 1.3)	0.221	1.0 (0.7,1.5)	0.929
LVGLS(%)	-	-	-	-	1.6 (1.0, 2.3)	**0.030**	-	-

Abbreviation: RVFAC: right ventricular fractional area change; TAPSE: tricuspid annular plane systolic excursion; RVFWLS: right ventricular free wall longitudinal strain; RVGLS: right ventricular global longitudinal strain; LVGLS: left ventricular global longitudinal strain.

Table S6. Univariable and multivariable Cox proportional hazards model for MACE prediction for segmental strain

**Segmental strain**	**Univariable model**		**multivariable model**	
	**HR (95%CI)**	**P-value**		
Free-wall basal (%)	1.1 (1.0, 1.2)	**0.007**	1.0 (1.0, 1.1)	0.207
Free-wall med (%)	1.1 (1.0, 1.2)	**0.015**	1.1 (1.0, 1.2)	0.196
Free-wall apical (%)	1.2 (1.1, 1.3)	**<0.001**	1.2 (1.1, 1.3)	**0.004**
Septum apical (%)	1.1 (1.0, 1.2)	0.058	-	-
Septum med (%)	1.1 (1.0, 1.2)	0.188	-	-
Septum basal (%)	1.0 (0.9, 1.1)	0.628	-	-

Table S7. Cut-off values for strain determined using ROC curve analysis

Test variables	Cutoff value	Sensitivity (%)	Specificity (%)
STE-RVFWLS(%)	-26	0.9	0.6
STE-RVGLS (%)	-24	0.9	0.7
STE-LVGLS (%)	-22	0.9	0.6

Abbreviation: RV-FAC: right ventricular fractional area change; TAPSE: tricuspid annular plane systolic excursion; STE: speckle-tracking echocardiography; RVFWLS: right ventricular free wall longitudinal strain; STE-RVGLS: right ventricular global longitudinal strain; LVGLS: left ventricular global longitudinal strain.

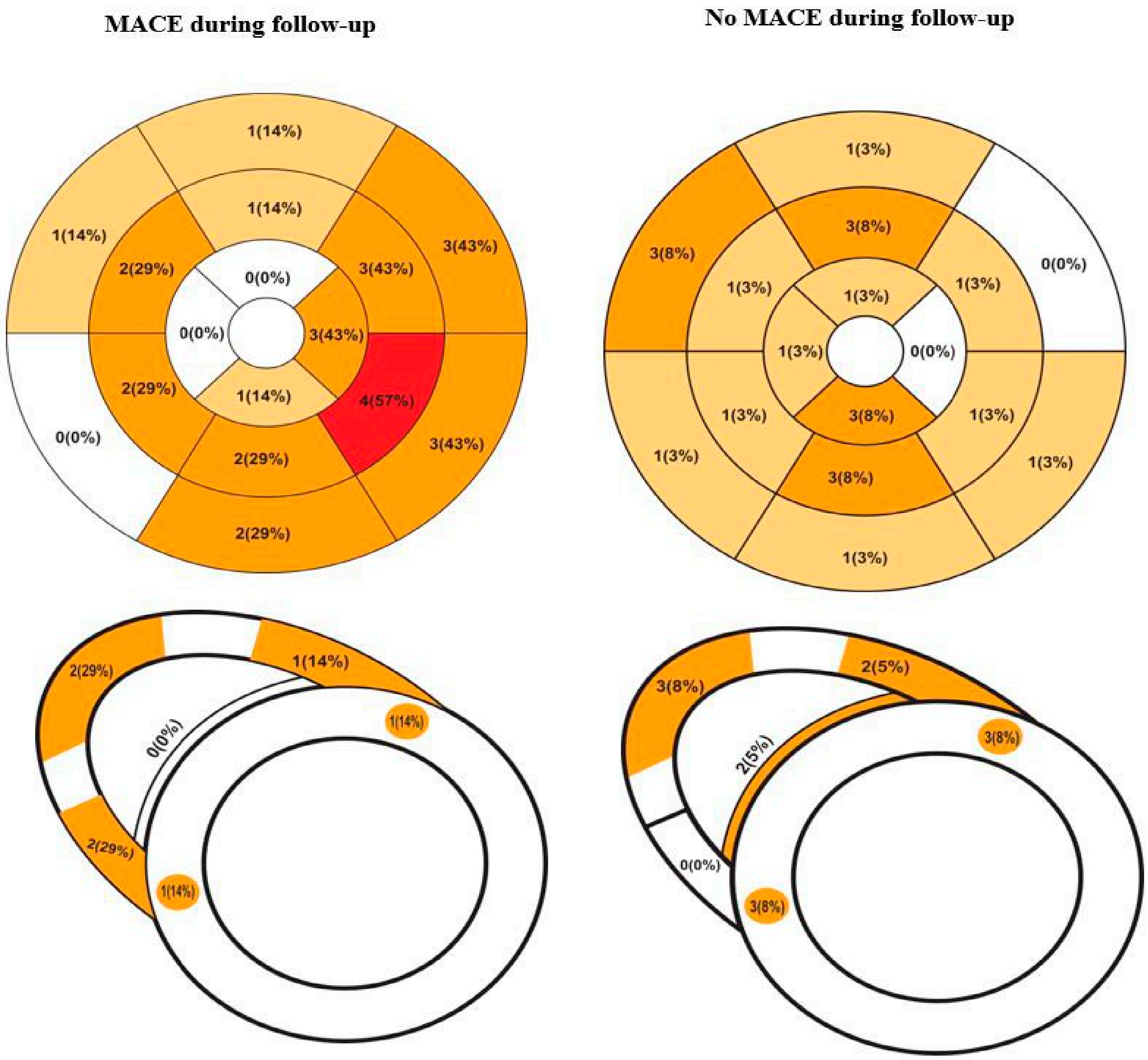

Figure S1. Right ventricular (RV) and left ventricular (LV) LGE distribution on CMR in patients with and without MACE during follow-up

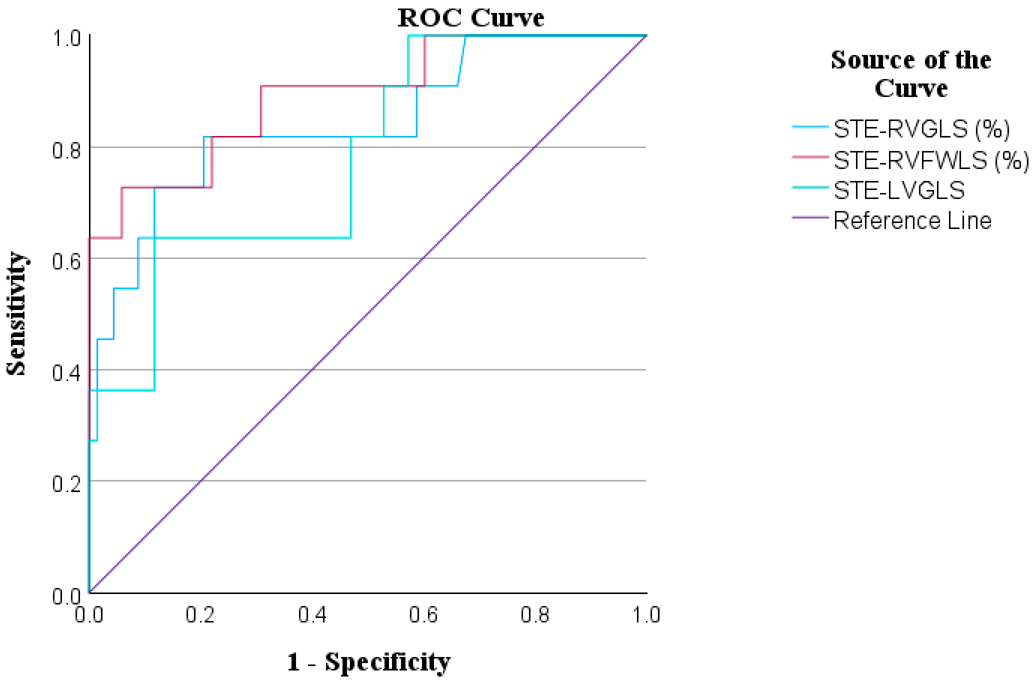

Figure S2. ROC curve, including all ARVC patients
